# Management of VEGFR-Targeted TKI for Thyroid Cancer

**DOI:** 10.3390/cancers13215536

**Published:** 2021-11-04

**Authors:** Tomohiro Enokida, Makoto Tahara

**Affiliations:** Department of Head and Neck Medical Oncology, National Cancer Center Hospital East, 6-5-1 Kashiwanoha, Kashiwa 277-8577, Japan; tenokida@east.ncc.go.jp

**Keywords:** thyroid cancer, vascular endothelial growth factor, tyrosine kinase inhibitor, adverse event

## Abstract

**Simple Summary:**

Anti-VEGFR therapy has become a mainstay of treatment for thyroid cancer across histological subtypes. However, the inhibition of this pathway is associated with particular adverse effects, some of which are life-threatening and may lead to the withdrawal of definitive treatment. To minimize this risk, the physician must recognize the characteristics of these adverse effects, including their timing and frequency, and adopt appropriate countermeasures. Moreover, management should more broadly encompass the appropriate subject selection for this treatment, as well as modification of the treatment schedule and consideration of alternative therapies for those patients harboring a risk of toxicity.

**Abstract:**

Recent advances in the development of multitarget tyrosine kinase inhibitors (MTKIs), which mainly target the vascular endothelial growth factor receptor (VEGFR), have improved prognoses and dramatically changed the treatment strategy for advanced thyroid cancer. However, adverse events related to this inhibition can interrupt treatment and sometimes lead to discontinuation. In addition, they can be annoying and potentially jeopardize the subjects’ quality of life, even allowing that the clinical outcome of patients with advanced thyroid cancer remains limited. In this review, we summarize the potential mechanisms underlying these adverse events (hypertension, proteinuria and renal impairment, hemorrhage, fistula formation/gastrointestinal perforation, wound healing, cardiovascular toxicities, hematological toxicity, diarrhea, fatigue, and acute cholecystitis), their characteristics, and actual management. Furthermore, we also discuss the importance of related factors, including alternative treatments that target other pathways, the necessity of subject selection for safer administration, and patient education.

## 1. Introduction

Thyroid cancer is the most prevalent endocrine cancer worldwide. Presently, four multitarget tyrosine kinase inhibitors (comprising sorafenib [1,2], Lenvatinib [3,4] vandetanib [5,6], and cabozantinib [7,8]) (MTKIs) are licensed as critical therapeutic options for the treatment of thyroid cancer, and have improved the progression-free survival (PFS) of patients in clinical trials and real-world studies. These compounds show activity against several receptor tyrosine kinases (RTKs), some involved in the pathogenesis of thyroid cancer (i.e., BRAF, RAS, RET) and others in the vascular angiogenic pathway (i.e., VEGFR2, platelet-derived growth factor (PDGFR)). These latter kinases—the main pro-angiogenic molecules in thyroid cancer—act by promoting the formation of a vast network of blood vessels. Accordingly, damaging the feeding blood vessels, especially vascular endothelium, appears to be the most important mechanism of action of the MTKIs in thyroid cancer. As these MTKIs are generally used as chronic therapies, it is important to effectively manage and minimize their toxicities and thereby enable patients who show benefit to continue treatment and obtain maximal clinical efficacy [9]. More particularly, the toxicity associated with VEGF pathway inhibition is common and has a rapid onset during the early phase of treatment, and—although this is rarely severe and life-threatening—patient quality of life (QOL) is nearly always affected. Accordingly, selecting the appropriate subject for this treatment is advised, with close clinical monitoring and proactive multidisciplinary management. Moreover, both physicians and patients should be educated to recognize drug-related toxicities to allow their early management. Physicians should also consider alternative therapeutic options that are consistent with the individual patient’s condition. Furthermore, despite the development of gene alteration-specific TKIs, such as BRAF-targeted ones, most patients who do not harbor these alterations are still candidates for the VEGFR-targeted TKI.

The main aim of this review is to summarize and discuss the mechanisms potentially underlying these adverse events (AEs) and our current understanding of the management of the side effects of MTKIs in thyroid cancer. We particularly focus on anti-VEGF-related mechanisms, with the aim of preventing their occurrence and exacerbation, and ideally of avoiding definitive drug withdrawal.

## 2. Adverse Effects of Anti-VEGFR Therapy and the General Principles of Their Management in Thyroid Cancer

AEs associated with VEGF pathway inhibition in thyroid cancer include hypertension, proteinuria, hemorrhage, fistula formation, cardiovascular adverse events and gastrointestinal perforation (GIP) (Table 1). Some of these conditions are rare but potentially life-threatening and may lead to treatment interruption and discontinuation. Post-marketing surveillance has revealed adverse events that were not found in clinical trials, owing to the increased number of patients receiving TKI treatment, including those subjects whose characteristics did not meet the inclusion criteria in the trials (e.g., renal adverse events rarely occurred in the phase III study, but they were found in daily practice [10]). It is recognized that each adverse event has a susceptibility period, but AEs generally occur early (as soon as 2–3 weeks after initiation) in treatment.

The median time to an adverse event of any severity grade in the SELECT trial, which evaluated lenvatinib in radioactive iodine (RAI)-refractory differentiated thyroid cancer (RR-DTC), was 12.1 weeks [11]. In particular, more patients in the older group (e.g., >65 years) experienced specific VEGF-related AEs of grade 3 or higher during MTKI treatment than did younger patients (e.g., lenvatinib-emergent hypertension: 49.1% vs. 36.8%, proteinuria: 13.2% vs. 7.7%, respectively). Furthermore, older patients were more likely to require dose interruption and reduction or to discontinue therapy in general [12]. Interestingly, several specific AEs were found to be predictive of a superior survival outcome. Among these, lenvatinib-emergent hypertension and diarrhea were associated with a PFS and overall survival (OS) advantage compared with patients treated with lenvatinib who did not experience these AEs [13]. Furthermore, different populations are characteristically predisposed toward AE risks with the same drug (e.g., the incidence of grade 3 or higher lenvatinib-emergent hypertension was 4.7% in an Italian real-life observational study in 94 DTC patients [14] and 80% in the Japanese population of SELECT [4]), suggesting the need to consider regional diversity regarding AE frequency and dose modification with TKIs.

**Figure 1:**
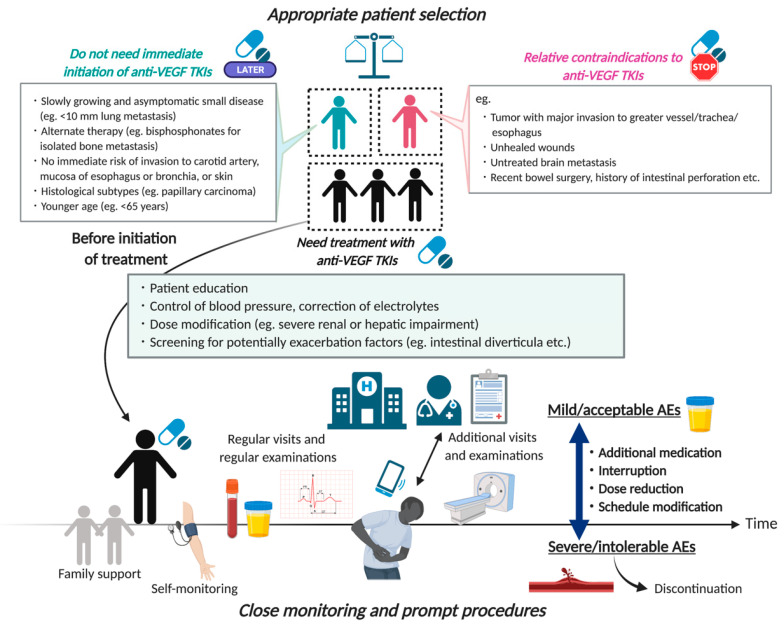
The concept of management of VEGFR-targeted TKI in thyroid cancer. AEs, adverse events.

As a general principle in the management of AEs, conservative, supportive medical care is applied for mild or moderate symptoms (common terminology criteria for adverse events (CTCAE): grade 1 and grade 2 events, respectively) and dose interruption for severe symptoms or those with life-threatening consequences (CTCAE grade 3 or grade 4 events, respectively), with subsequent consideration regarding restarting treatment at a lower dose (dose modification) upon the resumption of treatment once the adverse event has been resolved [15]. A very small number of severe and unacceptable VEGF-related AEs prevent the restart of treatment, including tumor-related fistula formation with severe hemorrhage (definitive withdrawal). Because the clinical outcome of patients with advanced thyroid cancer remains limited, adequate supportive care by healthcare workers for individual toxicity is strongly critical to improving the likelihood of efficacy and extending survival, while ensuring a good QOL during treatment. To ensure all treatment-related AEs are unerringly identified at the asymptomatic stage, regular examination in the clinic, including blood and urine tests and electrocardiograms, plays an important role. Moreover, considering that MTKI treatment for thyroid cancer is characterized by daily, long-term oral medication at home, patient education to aid in the early recognition of the signs and symptoms of AEs (e.g., self-monitoring of blood pressure) is essential to achieving early optimal management and intervention (Figure 1). The following section outlines the actual management in an item-by-item manner.

## 3. Appropriate Selection of Subjects and Optimal Timing of the Initiation of Treatment

Any consideration of the indications for VEGFR-targeted TKI in thyroid cancer must weigh the relative merits and demerits of VEGFR-targeted TKI. Careful subject selection at treatment initiation is one of the most important factors in the overall management strategy for AEs. First, given that life-threatening VEGF-related AEs could occur in any particular situation, physicians should properly recognize the relative contraindications of the drugs in advance and make an effort to minimize or avoid the risk of progression (Figure 1) [16]. These contraindications include existing active bleeding, substantial invasion into great vessels with a history of therapeutic external beam radiotherapy, transmural airway or esophagus invasion, and unhealed wounds. Any of these could result in a severe condition via the development of significant fistula and bleeding, as well as protracted wound healing. Bleeding itself is not an absolute contraindication to TKI treatment: the site of bleeding and its severity should be considered vis-à-vis therapy benefits in the evaluation of treatment discontinuation.

In patients with a rapidly growing tumor or metastasis close to the carotid artery, jugular vein, or hilus, the administration of MKIs should be carefully evaluated to avoid the risk of hemorrhage [17,18]. If the subject has safer alternative therapeutic options, the indication for MKIs must be evaluated. Specifically, surgery remains the cornerstone of treatment for locoregional recurrence. Other local therapies, including ethanol ablation, thermal ablation, chemoembolization, and external beam radiation therapy also effectively reduce recurrence [19,20]. If the metastatic tissue in patients with DTC remains sensitive to radioactive iodine, treatment with RAI should be considered [21]. Isolated bone metastases are treated with anti-osteolytic agents, either bisphosphonates or denosumab [19]. These procedures are used alone or in combination to try to avoid severe VEGF-related AEs. For patients harboring permanent and unsolvable risk factors, other systemic therapies, such as TKIs that do not target the VEGF pathway, can be considered instead, as described later.

The appropriate timing for the start of VEGFR-targeted TKI is also a critical management point. Except for anaplastic thyroid carcinoma (ATC), the tumor growth of thyroid cancers is generally slower than that of other cancers, even if the tumor becomes radioiodine-refractory. Indeed, patients with a tumor size of less than 1 cm will experience no symptoms and have a good quality of life. In contrast, toxicities related to VEGF-targeted TKI produce an overall deterioration in QOL in most patients. On balance, patients with an indolent disease do not immediately require tumor shrinkage by anticancer drugs at the expense of their QOL. To avoid this disadvantage and clarify the clinical meaning of the investigated drugs, the DECISION study, which evaluated sorafenib, and the SELECT study specified disease progression according to the RECIST criteria within 14 or 13 months as a requirement of study enrolment [1,3]. The National Comprehensive Cancer Network/American Thyroid Association guideline mentions that TKI treatment should be considered in “patients with metastatic, rapidly progressive, symptomatic, and/or imminently threatening disease” [22]. In this regard, approaches without close monitoring of the individual’s condition, namely, by imaging-based examination, may increase the risk of invasion into a vital structure, such as a carotid artery, and may lead to the relatively contraindicated situation described above. On the other hand, even among patients with neither rapidly progressive nor symptomatic disease, some will require the immediate use of a VEGF-targeted TKI. A sub-analysis of the SELECT study suggested that the watch-and-wait approach might worsen outcomes in older patients (>65 years) [12], in those with follicular thyroid cancer (FTC) (the OS was significantly better in the lenvatinib arm than the placebo arm among those with FTC (hazard ratio (HR) 0.41, 95% confidence interval (CI) 0.18–0.97; *p* < 0.035) [23]), and those with lung metastases of ≥ 1.0 cm [24]. These findings indicated that the delayed use of MTKIs worsens patient outcomes in specific populations, irrespective of the presence and absence of symptoms. The ongoing international, prospective, open-label, multicenter, non-interventional RIFTOS MKI study is now investigating the time period to symptomatic progression from study entry in asymptomatic patients with progressive RR-DTC, and should aid in the establishment of evidence-based guidelines for the optimal timing of lenvatinib and sorafenib treatment initiation in asymptomatic patients with RR-DTC (Clinical trial: 02303444) [25,26,27,28].

## 4. Management of Individual AEs

### 4.1. Hypertension

Hypertension is the most frequently observed AE that is associated with TKIs that inhibit VEGF, particularly those inhibiting VEGFR2. Anti-VEGF therapies inhibit VEGF-mediated vasodilation via the activation of nitric oxide (NO) synthase at the level of vascular endothelium [29]. In the SELECT study, the median time to development of hypertension was 2.3 weeks (range: 1.4–5.0) [13], versus eight days in Japanese patients [30]. Treatment-emergent hypertension was associated with a 5.9-month median progression-free survival advantage (HR 0.59, 95% CI 0.39–0.88; *p* = 0.009) [13]. The degree differed according to the drug; in a systematic review and meta-analysis of seven studies regarding TKIs for advanced or RR-DTC, patients treated with sorafenib had a lower frequency of both all-grade and grade ≥3 hypertension (41.6% and 10.5%) than those treated with lenvatinib (65.2% and 35.2%); the differences were statistically significant [31].

**Figure 2:**
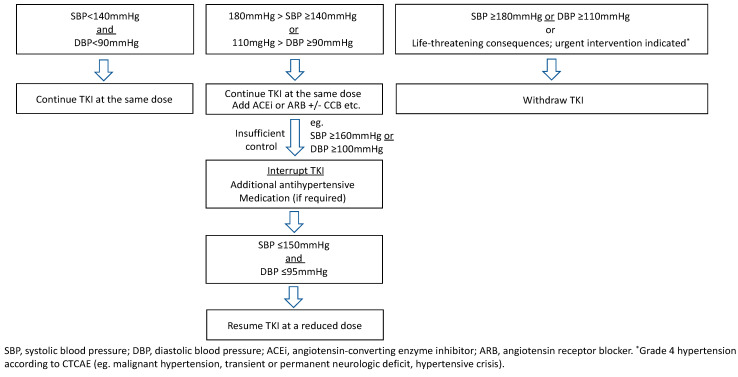
Proposal of management of VEGFR-targeted TKIs-induced hypertension.

Because anti-VEGFR TKI therapies can lead to the new onset or worsening of established hypertension, all patients should have optimal blood pressure (BP) (<140/90 mmHg) control before the initiation of treatment and maintain a controlled BP (<140/90 mmHg, or lower in the case of overt proteinuria) throughout treatment. The use of 24-hour BP monitoring can detect early development and accurately assess BP changes in patients treated with anti-VEGFR TKI [32]. BP measurement at the same time in the morning at least once a day at home should be recommended. Once hypertension (>140/90 mmHg) or an increase in diastolic BP greater than 20 mmHg over baseline has developed [33], angiotensin-converting enzyme inhibitors (ACEi) or angiotensin II receptor blockers (ARB) should be considered first, followed by calcium channel blockers, diuretics/thiazides, and β-adrenoceptor blockers if required, either as monotherapy or in combination [34,35,36]. For patients with persistent proteinuria, in the absence of a specific therapy directed against the underlying disease, a decrease in intraglomerular pressure, which might reduce protein excretion, may be achieved by administering an angiotensin-converting enzyme inhibitor or angiotensin receptor blocker (offering a renoprotective effect). ACEi and beta-blockers are the preferred antihypertensive drugs in patients with or at risk of heart failure/left ventricular dysfunction [35]. On the other hand, the use of diuretics may raise the risk of electrolyte depletion and consequent QT prolongation, and should therefore not be considered for first-line therapy because of potential dehydration due to concomitant diarrhea, nausea, or vomiting [35]. Care is required, especially in patients treated with vandetanib, which potentially causes diarrhea and QT prolongation. TKI should be interrupted in patients with resistant hypertension (≥ 160/100 mmHg) despite antihypertensive therapy until the blood pressure drops to a normal range, and then restarted at a lower dose level. If the patient developed severe hypertension (e.g., ≥ 180/110 mmHg), the TKIs should be withdrawn (Figure 2).

### 4.2. Proteinuria and Renal Impairment

The mechanism underlying the proteinuria associated with VEGF inhibitors is unclear. Possible explanations include thrombotic microangiopathy, which impairs the VEGFR-expressing podocytes that play a central role in glomerular filtration [37,38,39], and glomerulopathies such as minimal change disease and focal segmental glomerulosclerosis. A review of anti-VEGF renal side effects revealed that the most common renal side effect of anti-VEGF drugs is proteinuria, ranging from 21% to 63%, and that it frequently occurs in association with hypertension [40]. Other meta-analyses showed incidences of 18.7% for all grades of proteinuria and 2.4% for high-grade proteinuria in patients receiving VEGFR-targeted TKIs. However, these meta-analyses did not include any studies with lenvatinib. In the SELECT study, approximately one-third of all patients developed proteinuria of any grade, and 10% experienced grade ≥ 3 proteinuria [41]. In a subgroup analysis of the Japanese population in the SELECT trial, the incidence of renal adverse effects was higher, with any-grade proteinuria of 63.3% and grade 3 proteinuria of 20%, even after the dosage had been adjusted for weight [4]. Although the DECISION study did not report on sorafenib-associated renal adverse effects [1], real-world experience with lenvatinib and sorafenib in Japanese populations showed much higher incidences of proteinuria of any grade, namely 60.8% and 27.8%, respectively [42]. Although glomerular injury can precede the new development of hypertension, patients with renal dysfunction caused by other comorbidities at baseline, such as hypertension and diabetes, should be carefully managed. Onset is generally early (median time 6.1 weeks in SELECT [11]) but asymptomatic, and accurate monitoring by regular urinalysis, possibly with timely drug discontinuation, should therefore be conducted.

Evidence-based guidelines for the management of VEGFR-targeted agent-induced proteinuria are lacking. For lenvatinib-induced proteinuria, lenvatinib may be continued if proteinuria is grade 1 or 2, based on the criteria set in clinical trials. In the previous studies, treatment interruption was mandatory when proteinuria reached grade 3 (urinary protein ≥ 3.5 g/d or a urine protein to creatinine ratio ≥ 3.5) [3,4,43]. While proteinuria itself is rarely life-threatening (i.e., the degree of proteinuria did not significantly correlate with renal dysfunction, defined by a decrease in the estimated glomerular filtration rate (GFR)) [42], it is not realistic to apply these criteria universally, and physicians must balance treatment benefits versus the potential harms of toxicity. In this regard, urinalysis by a combination of the dipstick test and the urine protein:creatinine ratio (UPCR) showed promise in preventing unnecessary lenvatinib interruption in patients with advanced thyroid cancer, by eliminating the overestimation of proteinuria that occurs with qualitative dipstick urinalysis only [44].

If grade 1 or 2 proteinuria occurs in high-risk patients with edema, fluid collection, or elevated serum creatinine, treatment should be interrupted. Lenvatinib may be continued at the same dose if the urinary protein is < 3.5 g/day and there is no edema, fluid collection, or elevation in serum creatinine. After the proteinuria has recovered or improved to a lower grade, lenvatinib treatment may be restarted at a reduced dose. Although discontinuation of the anti-VEGF agent results in a significant reduction in proteinuria, persistence is common [45]. Furthermore, the prescribing of diuretics for edema and a statin for hyperlipidemia are recommended. [46].

In the SELECT trial, the incidence of acute renal failure was 4%, and that of grade ≥ 3 was 1.9% [3]. Gastrointestinal toxicity, including nausea, vomiting, and loss of appetite, are the primary risk factors for renal toxicity: the administration of diuretics for hypertension or fluid retention might cause their exacerbation, and physicians thus need to pay attention when prescribing these medicines. Besides, given the safety evidence regarding the renal toxicity of sorafenib in various cancer types, including renal cell carcinoma, the drug can be safely given in patients with mild and moderate renal insufficiency [42,47,48]. Renal insufficiency and diabetes insipidus have been reported in clinical trials of vandetanib for medullary thyroid cancer, although causation has not been established [5,49].

### 4.3. Hemorrhage

Because of its strong anti-VEGFR activity, all antiangiogenic MKIs carry a risk of bleeding, presumably due to blood-vessel destabilization following decreased matrix deposition, as well as the loss of vascular integrity, resulting in blood vessel rupture and thrombocytopenia [9,50]. Hemorrhage most commonly manifests as epistaxis of mild severity. However, if the tumor mass is severe and vital neck structures are involved, like a major artery, the trachea and esophagus, the extensive necrosis caused by antiangiogenic tyrosine kinase inhibitor therapy could lead to potentially life-threatening AEs, including a rupture of the carotid artery, tracheoesophageal fistula and esophageal perforation [11,51]. In the ZETA study, which evaluated cabozantinib in progressive medullary thyroid cancer, 2 of the 219 patients treated with cabozantinib developed grade 5 bleeding, of which one instance was recognized as treatment-related [5,6]. In the SELECT study, 35% of lenvatinib-treated patients experienced hemorrhagic events, compared with 18% of the placebo group [3]. Moreover, 14 cases of hemorrhage were reported in a post-marketing study for lenvatinib in Japan [52]. Most cases appeared to be associated with tumor shrinkage and necrosis surrounding the carotid artery. Incidence might have been influenced by histological subtype: 3.4% of patients with locally invasive ATC (8 of 238 cases) and 0.8% with DTC (6 of 778 cases) developed hemorrhagic events [46]. In addition, the influence of a history of prior treatment with radiotherapy was noted: seven cases had received external radiation. Considering the relatively high rate of a history of radiotherapy, it is uncertain whether radiation therapy should be prioritized over systemic therapy to achieve local disease control. Bleeding events usually occurred within a few months after the initiation of MTKIs; accordingly, diagnostic imaging should be considered monthly for at least the first several months after initiation, to check the anatomical relationship between the tumor and vital organs. Besides this, patients taking antiplatelet drugs and anticoagulants who develop thrombocytopenia due to the VEGFR-targeted TKIs might be at further increased risk of bleeding, and those with a history of inflammatory bowel disease or diverticulitis should be monitored carefully for gastrointestinal bleeding. TKIs should be withheld in patients who develop a grade 3 hemorrhage until resolution to grade 0 or 1 [53,54]; once resolved, lenvatinib can be resumed at a reduced dose or discontinued, depending on the severity of the hemorrhagic event. Lenvatinib should be discontinued in patients who experience a grade 4 hemorrhagic event [53,54].

### 4.4. Fistula Formation and Gastrointestinal Perforation

Fistula formation is an uncommon but occasionally life-threatening antiangiogenic TKI-associated AE [7,55,56]. As with hemorrhage, particular attention should be paid to the radiotherapy prescribed to the lesion, prior to surgery, or cases where the tumor invades crucial neck structures [56]. Thyroid cancer patients generally undergo such loco-regional procedures before TKI therapy, thus increasing the risk of fistula. Delayed wound healing due to the administration of antiangiogenic TKIs is plausible, and physicians sometimes experience fistula formation together with a favorable clinical response to TKI [57]. In one study, the trachea and esophagus were involved in 37% and 21% of patients with invasive thyroid cancer, respectively [58], indicating that tracheoesophageal fistula (TEF) formation can occur during antiangiogenic therapy. Known risk factors for TEF formation include the characteristics of the tumor (e.g., extension into the mediastinum) and local treatment history (e.g., external beam radiation) [55,56]. Another morbid condition is esophageal perforation, which has a 40–60% mortality rate when treatment is delayed [59]. In the SELECT trial, fistula formation occurred in 1.5% of patients receiving lenvatinib, with 0.8% experiencing a grade 3 or worse event [3]. In the abovementioned post-marketing study, in contrast, 11 patients presented with fistula formation, with the occurrence of a small hemorrhage being predictive in 7 cases [52]. Again, particular attention is required when prescribing VEGF-targeted TKIs after radiotherapy [19,56]. Furthermore, in a retrospective analysis of 111 thyroid cancer cases (79 RR-DTC and 32 ATC) in a single institution in Japan, 11 patients with ATC (34.4%) and 7 with DTC (8.9%) developed skin fistula. The mortality rate among these patients was 38.9% (7/18), including three deaths caused by major bleeding and four attributable to mediastinitis or pneumonia [60]. In the ZETA study, 3 out of 219 patients treated with cabozantinib developed a treatment-related grade 5 fistula [5,6].

Anti-VEGF therapies also contribute to the development of GIP. VEGF inhibition is considered to perturb platelet–endothelial cell interactions, which can lead to a loss of vascular integrity and submucosal inflammation [61,62]. In some cases, GIP might be associated with the exacerbation of preexisting ulcers or diverticulitis, tumor shrinkage as a result of treatment, or a recent history of sigmoidoscopy or colonoscopy [9,62,63]. Thus, systematic gastrointestinal screening for lesions that have the potential to become risks should be advised before the initiation of treatment, especially in patients with iron deficiency anemia of unknown origin [63]. In addition to these, pathologically proven intestinal metastasis-related GIP has been reported in lenvatinib-treated patients, namely, anaplastic thyroid cancer and hepatocellular carcinoma [64].

The management of fistula, especially when it involves the gastrointestinal tract, and GIP includes fasting and bowel rest with total parenteral nutrition, broad-spectrum antibiotics, and surgical procedures (e.g., resection of the affected bowel) if required [50,65,66]. It is important to note that surgical intervention in patients treated with antiangiogenic therapies can be complicated by impaired wound healing [50]. VEGFR-targeted TKI-treated patients who develop fistulas or GIP should discontinue therapy [53].

### 4.5. Wound Healing

Antiangiogenic TKIs are associated with wound-healing complications, including the reopening of previously healed wounds [9,50,67,68,69,70,71,72,73]. As angiogenesis is required to maintain vascular integrity, epithelialization, and wound strength, the inhibition of this process can delay or impair wound repair, particularly after surgery [9,50,61,67,74]. In addition to that, the strong fibroblast growth factor (FGF) inhibition that is particularly obtained by lenvatinib in this field might also contribute to the adverse event [75]. Thus, these therapies typically require the temporary discontinuation of the drug before major surgery [67]. Although there are no prospective data, antiangiogenic TKIs should be withheld for 3–5 times the half-life of the drug involved (e.g., the half-life of lenvatinib and sorafenib is 35.4 h and 28.1 h, respectively), or ideally for one week prior to major surgery, and all should be withheld until the wounds are at least reasonably healed [54]. For example, post-marketing surveillance revealed the incidence of pneumothorax during lenvatinib therapy, especially in patients with lung metastasis. On the other hand, a retrospective study of surgical interventions in thyroid cancer patients undergoing lenvatinib treatment reported no primary wound complications, and only a single case of delayed healing secondary to the placement of a thoracic drain for acute pneumothorax (57.1% were performed without the withdrawal of lenvatinib before the procedure, and 50% reintroduced lenvatinib just after the procedure) [76]. Given the potential for precipitous disease progression (flare) after the interruption/discontinuation of TKIs, probably due to the rapid regeneration of tumor vessels, further understanding of the appropriate duration of drug withholding is essential to minimize the risk safely [77,78].

### 4.6. Cardiovascular Toxicities

In addition to hypertension, as described above, it has now been clearly demonstrated that anti-VEGF agents exhibit various cardiotoxic manifestations, including cardiac dysfunction, arterial and venous thrombosis, and QTc prolongation [79,80], and that these are some of the most challenging events for patients. Therefore, a baseline assessment to identify risks, to guide clinicians toward safer management, should be considered before treatment. Once the risk factors (e.g., uncontrolled hypertension, electrolyte imbalances) and other related complications are recognized, they should be treated and/or corrected prior to therapy and closely monitored during antitumor therapy [81]. To this end, a multidisciplinary team that includes both oncologists and cardiologists (cardio-oncology) would play a vital role, as needed.

The inhibition of VEGF or PDGF may cause cardiomyocyte cell death and prevent cardiac remodeling, resulting in cardiac dysfunction (congestive heart failure) [82,83]. Across clinical trials in 799 patients with DTC, renal cell carcinoma (RCC), and hepatocellular carcinoma (HCC), cardiac dysfunction of grade 3 or higher occurred in 3% of lenvatinib-treated patients [84]. In the EXAM study, which evaluated cabozantinib for medullary thyroid cancer (MTC), one treatment-related grade 5 cardiopulmonary failure was observed out of 219 cabozantinib-treated patients [7,8]. Management of heart failure should include the careful monitoring and administration of routine heart failure therapies [9,53]. In addition to the baseline, patients undergoing VEGFR inhibitor therapy should undergo an echocardiogram after the first month of therapy and then every three months thereafter [85]. The appropriate management of hypertension within the normal range, using beta-blockers and ACEi/ARB and diuretics for patients with fluid overload/edema, could reduce cardiac load [9,53,86,87]. Lenvatinib should be withheld for grade 3 cardiac dysfunction until resolution to grade 0 or 1. Upon resolution, lenvatinib can be resumed at a lower dose or discontinued, depending on disease severity. If lenvatinib is resumed, BP should be monitored daily and maintained within the normal range. Lenvatinib should be discontinued in grade 4 cardiac dysfunction [53].

Vascular endothelial death by the inhibition of VEGF can result in the exposure of procoagulant phospholipids on the luminal plasma membrane and underlying extracellular matrix, as well as a tendency to thrombosis [88]. Inhibition may also lead to the overproduction of erythropoietin in the liver, which increases hematocrit and blood viscosity [89,90]. In fact, an increased incidence of high-grade arterial thrombotic events has been reported (Peto odds ratio, 4.72, 95% CI: 1.18–18.95; *p* = 0.029), including myocardial infarction and cerebrovascular events, in 1781 patients with advanced thyroid cancer who were undergoing TKI therapy [91]. In addition to arterial thrombosis, venous thrombosis, including pulmonary embolisms, was reported in trials evaluating cabozantinib [7] and lenvatinib [3]. Once these events are detected, the basic approach should be antiplatelet therapy for arterial thrombosis or anticoagulation for venous thrombosis, together with the cessation of MTKI [53,87]. TKIs can be maintained during anti-VEGF therapy if the clinical treatment benefits outweigh the risks of complications.

QTc reflects the total duration of ventricular activation and recovery [92], and QTc prolongation is a substantial adverse effect and a high-risk factor for sudden death. Given that torsades de pointes (TdP) rarely occurs when the QTc is < 500 ms [86], QTc prolongation > 500 ms (and deltaQT (change from baseline) of > 60 ms) is considered a particular concern. The downregulation of phosphatidylinositol-3 kinase (PI3K), signaling directly or indirectly via tyrosine kinase inhibition, prolongs the QT interval by affecting multiple ion channels [93]. Although lenvatinib exerts no clinically meaningful effect on the QTc interval in healthy volunteers [94], the overall grade and grade ≥ 3 QTc prolongation caused by lenvatinib administration in thyroid cancer was noted in 8% and 1.5% of patients, respectively [3]. Overall, 14% of thyroid cancer patients treated with vandetanib had some QTc prolongation, and a severe lengthening of the QTc interval occurred in 8% of patients [5,6]. Interestingly, no significant difference in relative risk according to the duration of treatment was seen in trials with short (relative risk (RR) 11.3, 95% CI: 4.4–29.0) vs. long duration (RR.8.21, 95% CI: 3.51–19.2) [95]. Vandetanib should not be given to patients with congenital long QTc syndrome or a history of TdP unless all risk factors that contribute to the TdP are corrected. The echocardiogram (ECG), serum potassium, calcium and magnesium levels, and thyroid-stimulating hormone (TSH) levels should be evaluated, monitored and corrected as necessary before and throughout MTKI therapy, the latter because an increase in QTc is directly related to TSH level—as in overt hypothyroidism and hypothyroidism—and also causes hypocalcemia [54,96]. The ECG, serum potassium, calcium, and magnesium levels, and TSH level should also be measured at 1, 3, 6, and 12 weeks after initiation or dose changes, for example, and every three months thereafter for at least a year [54,96,97]. Moreover, it is necessary to avoid the concurrent use of possible inhibitors of cytochrome P450 3A4 (CYP3A4), which may increase the plasma concentration of many of the antiangiogenic TKIs. Diuretic use implies the risk of electrolyte depletion and consequent QT prolongation; therefore, it should not be used as first-line therapy for hypertension because of potential dehydration due to concomitant diarrhea, nausea, or vomiting [35]. A general recommendation from both the US Food and Drug Administration and European Medicines Agency is to temporarily interrupt treatment in the case of QTc prolongation above 500 ms (or if QTc prolongation is > 60 ms above the baseline). In such cases, electrolyte abnormalities should be corrected and cardiac risk factors for QT prolongation should be controlled [35]. Treatment can then be resumed at a reduced dose once the QTc normalizes (<480 ms) [35]. Higher doses of vandetanib were associated with increased risk (RR 10.60 against 4.83 for lower doses) [95].

### 4.7. Hematological Toxicity (Thrombocytopenia)

VEGF receptors are expressed and play different roles in the commitment, differentiation, proliferation, survival, and polyploidization of hematopoietic stem cells (HSCs)/megakaryocytes (MKs). They do this via autocrine, paracrine, and/or even intracrine loops [98]. MTKIs inhibit the VEGF and PDGF pathways on hematopoietic stem cells, leading to cytopenia, especially when it occurs together with thrombocytopenia [99,100,101]. A meta-analysis of 3221 patients treated with sorafenib revealed incidences of sorafenib-associated all-grade and high-grade thrombocytopenia of 25.3% and 4.0%, respectively [102]. At the same time, grade ≥ 3 thrombocytopenia was observed in 25.4% of the patients in a meta-analysis of lenvatinib trials [103].

Thrombocytopenia increases the risk of bleeding, particularly in patients with grade 4 thrombocytopenia and/or the concurrent use of antiplatelets and anticoagulants. Accordingly, complete blood counts should be carefully and routinely monitored throughout treatment [9,103]. When grade 3 or 4 thrombocytopenia occurs, MTKI administration should be interrupted, then resumed upon the recovery of platelet numbers at a reduced dose. Generally, thrombocytopenia improves rapidly after drug interruption [46].

### 4.8. Diarrhea

The mechanism of small molecule-TKI–induced diarrhea remains under investigation. Given that VEGFR and epidermal growth factor receptor (EGFR) are both highly expressed in the gut and that diarrhea is more frequent with the more common multi-kinase inhibitors targeting both VEGF and EGFR, such as vandetanib or sorafenib, compared with pure VEGFR inhibitors, the inhibition of both pathways might contribute to lowered cell proliferation and reduced capillary networks in the intestinal villi, resulting in diarrhea [104,105]. Interestingly, the occurrence of MTKI-induced diarrhea relates to treatment success. Among the associations with OS in the multivariate model of the SELECT trial, the occurrence of diarrhea was identified as an independent predictive factor for a favorable OS (HR 0.55, 95% CI: 0.33–0.92; *p* = 0.023), along with a baseline Eastern Cooperative Oncology Group performance status scale (ECOG PS) and histology [11]. The median time to the first onset of lenvatinib-induced diarrhea was 12.1 weeks [11]. Although diarrhea is generally mild (grade 1 or 2) and manageable with antidiarrheal agents, including loperamide, proper management is needed to avoid undesirable secondary events, such as electrolyte depletion and related QT prolongation, and renal impairment due to dehydration, especially in patients taking diuretics as well [104]. Treatment should be interrupted for grade 3 or 4 diarrhea, and subsequent dose reductions may be necessary when treatment is resumed. In addition to these side effects, pancreatic atrophy has also been reported in patients receiving long-term sorafenib, and physicians should consider this possibility in patients treated with sorafenib who develop refractory diarrhea [106].

### 4.9. Fatigue

Fatigue during anti-VEGF is likely to be multifactorial and is difficult to distinguish from cancer-related symptoms. Contributing treatment-related factors may include anemia, dehydration, electrolyte imbalance that is secondary to diarrhea and gastrointestinal toxicity, cardiac dysfunction as described above, and thyroid dysfunction. The inhibition of VEGFR might lead to a subsequent increase in TSH level due to any one of several mechanisms, including destructive thyroiditis [107]. In another study, 59% of patients treated with lenvatinib in the SELECT trial experienced fatigue, with 9.2% at grade ≥ 3, while TSH levels above 0.5 mU/L were observed in 57%, compared to 14% of patients on placebo [3,53]. The median time to the first onset of lenvatinib-induced fatigue was three weeks [11]. Treatment interruption and dose modification should be addressed if a patient complains of moderate to severe fatigue even after the correction of treatable factors.

### 4.10. Acute Cholecystitis

Acute cholecystitis has been reported as an adverse event associated with anti-VEGFR TKIs across tumor types [108,109,110,111,112,113], but its mechanism remains unclear. One candidate is microvascular ischemia and imbalance in stress adaptation, via the inhibition of VEGF signaling in cholangiocytes expressing VEGFRs. In the SELECT study, only one case (0.2%) of grade 3 acute cholecystitis was reported [3]; however, at least 11 cases of acute cholecystitis have been reported among thyroid cancer patients treated with lenvatinib in Japan since the drug was approved. Upon investigation, the Pharmaceuticals and Medical Devices Agency (PMDA) concluded that this adverse event should be added to the clinically significant adverse reactions section [114]. Nervo et al. also reported five patients (14.7%) treated with lenvatinib for progressive RR-DTC, excluding those who underwent cholecystectomy before the start of therapy, and who developed symptomatic, radiologically confirmed biliary disease after a median time of 4.4 months of lenvatinib treatment and thus underwent cholecystectomy [115]. Physicians should be aware of this adverse effect, primarily when patients complain of upper abdominal pain and particularly in those with a history of gallbladder stones or other biliary tract problems [57].

## 5. Other Factors for Appropriate Management of Anti-VEGFR TKIs Therapy

### 5.1. Patient Education and Institution Infrastructure

In addition to adequate supportive care and proper treatment interruption, dose modification, and discontinuation for each toxicity as described above, patient education concerning the risks and benefits of TKI treatment is essential for the early recognition of adverse events (e.g., self-monitoring BP to detect early changes that might be missed during sporadic clinic visits) and their optimal early-phase management by medical providers. At the same time, the hospital/institute must accept calls from patients 24 h a day, every day.

### 5.2. Alternative Schedules and Initial Dose of the Drug

A post hoc analysis of data from the SELECT trial showed that the prolonged interruption of lenvatinib (>10% of the total treatment duration) could impair efficacy compared with minimal treatment interruptions (<10%), albeit that this interrupted regimen was still more effective than the placebo [116]. On the other hand, our cohort studies of RR-DTC patients treated with lenvatinib demonstrated that progression-free survival, time to treatment failure, and overall survival were significantly longer in patients who used planned drug holidays, namely, dose interruptions in accordance with the timing of severe or intolerable adverse events, than in those who did not [117,118]. Although a prospective assessment is needed, this strategy could avoid treatment withdrawal, dose modification, and—most importantly—definitive discontinuation that eventually leads to tumor regrowth. With respect to the initial dose, it is recommended that patients with severe hepatic impairment start lenvatinib at 14 mg once daily instead of at 24 mg once daily [119]. Nevertheless, there is no evidence showing maintained efficacy and reduced toxicity in RR-DTC patients treated with VEGFR-targeted TKI that is started at a reduced dose. A randomized phase II clinical trial which investigated if a lower starting dose of lenvatinib of 18 mg yielded comparable efficacy to 24 mg in patients with RR-DTC did not demonstrate the noninferiority of 18 mg vs. 24 mg based on the overall response rate as of week 24 (40.3% vs. 57.3%) [120].

### 5.3. Consideration of Other Systemic Drugs Not Targeting VEGF or with a Different Toxicity Profile

When patients have experienced or are at high risk of severe side-effects with VEGF-targeting effect, it is reasonable to switch from anti-VEGFR TKIs to other systemic therapies that either do not directly target VEGF or have a different toxicity profile. For example, BRAF-, RET- and neurotrophic receptor kinase (NTRK)-directed therapy may be preferred options for patients harboring a BRAF mutation, or with RET- and NTRK-altered disease with a high risk of bleeding [121,122,123,124,125,126]. In addition, even when prescribing anti-VEGFR TKIs, physicians can select drugs with equivalent efficacy but different toxicity profiles in accordance with the patient’s condition. Although bleeding and thrombosis are more common with cabozantinib, for example, this drug might be preferred over vandetanib, especially in view of the increased frequency of long-QT interactions with the latter.

## 6. Conclusions

The toxicity profile of anti-VEGFR TKIs in the treatment of advanced thyroid cancer is well understood, and evidence for their management is accumulating. To provide the best anti-tumor efficacy and more prolonged survival while maintaining individual QOL, the physician should be closely aware of toxicities, undertake appropriate procedures, and decide on treatment interruption, dose modification, and discontinuation as needed. In a broad sense, proper selection among treatment candidates and the consideration of alternative treatment options in place of VEGF-targeted therapy for patients at high risk of intolerable anti-VEGF-related toxicities are manifestations of good management. Furthermore, comprehensive patient education is essential for early detection and the start of optimal care.

## Figures and Tables

**Table 1:** AEs associated with VEGFR inhibition in phase III trials for advanced thyroid cancer.

**Drug (Study)**	**Type of Cancer**	**No. of Patients**	**% of Selected Adverse Events {≥Grade 3} (Placebo)**					
			**Hypertension**	**Proteinuria**	**Renal Impairment/Failure**	**Hemorrhage**	**Fistula Formation**	**Wound Complication**
Sorafenib [1,2] (DECISION)	DTC	419	40.6% {9.7%} (12.4% {2.4%})	NR	NR	NR	NR	NR
Lenvatinib [3,4] (SELECT)	DTC	392	67.8% {42%} (9.2% {2.3%})	31.0% {10.0%} (1.5% {0%})	4.2% (1.9%)	NR (1 case of Gr5 probably treatment-related hemorrhagic stroke)	GI fistula: 1.5% {0.8%}	Wound dehiscence: NR {0.4%}
Vandetanib [5,6] (ZETA)	MTC	331	32% {9%} (16% {0%})	NR	NR	NR	NR	NR
Cabozantinib [7,8] (EXAM)	MTC	330	32.7% {8.4%} (4.6% {0.9%})	1.9% {0.9%} (0% {0%})	NR	25.2% {3.3%, 1 case of Gr5 treatment- related hemorrhage (15.6% {0.9%})	GI fistula: 0.9% {0.5%} (0% {0%}) Non-GI fistula: 3.7% {1.9%} (0% {0%})	1.9% {0.9%} (0.9% {0%})
**Drug** **(Study)**	**Type of** **Cancer**	**No. of** **Patients**	**% of Selected Adverse Events {≥** **grade 3} (Placebo)**					
			**Heart Failure**	**Thrombosis**	**ECG QT ** **Prolonged**	**Thrombocytopenia**	**Diarrhea**	**Fatigue**
Sorafenib [1,2] (DECISION)	DTC	419	NR	NR	NR	NR	68.6% {5.8%} (15.3% {1%})	49.8% {5.8%} (25.4% {1.4%})
Lenvatinib [3,4] (SELECT)	DTC	392	7% {2%}	ATE: 5.4% {2.7%} VTE: 5.4% {3.8%} PE: 2.7% {2.7%}	8% {1.5%}	8.8% {1.5%} (1.5% {0%})	59.4% {8.0%} (8.4% {0%})	59.0% {9.2%} (27.5% {2.3%})
Vandetanib [5,6] (ZETA)	MTC	331	NR	NR	14% {8%} (1% {1%})	NR	56% {11%} (26% {2%})	24% {6%} (23% {1%})
Cabozantinib [7,8] (EXAM)	MTC	330	NR {1 case of Gr5 treatment-related cardiopulmonary failure}	ATE:2.3% {0.9%} (0% {0%}) VTE:5.6% {3.7%} (2.8% {1.8%})	0%	35% {0%} (4% {3%})	63.1% {15.9%} (33% {1.8%})	40.7% {9.3%} (28.4% {2.8%})

DTC, differentiated thyroid cancer; MTC, medullary thyroid cancer; NR, not reported; ECG, electrocardiogram; GI, gastrointestinal; ATE, arterial thromboembolism; VTE, venous thromboembolism; PE, pulmonary embolism. The number in the bracket indicates the incidence of AE with grade 3 or more. The number in parentheses indicates the incidence of AE observed in the placebo arm in each trial.

## References

[1] Brose M.S., Nutting C.M., Jarzab B., Elisei R., Siena S., Bastholt L., de la Fouchardiere C., Pacini F., Paschke R., Shong Y.K. (2014). Sorafenib in Radioactive Iodine-Refractory, Locally Advanced or Metastatic Differentiated Thyroid Cancer: A Randomised, Double-Blind, Phase 3 Trial. Lancet.

[2] Brose M., Jarzab B., Elisei R., Giannetta L., Bastholt L., Fouchardiere C., Pacini F., Paschke R., Nutting C., Shong Y.K. (2016). Final Overall Survival Analysis of Patients with Locally Advanced or Metastatic Radioactive Iodine-Refractory Differentiated Thyroid Cancer (RAI-rDTC) Treated with Sorafenib in the Phase 3 DECISION Trial: An Exploratory Crossover Adjustment Analyses. Ann. Oncol..

[3] Schlumberger M., Tahara M., Wirth L.J., Robinson B., Brose M.S., Elisei R., Habra M.A., Newbold K., Shah M.H., Hoff A.O. (2015). Lenvatinib versus Placebo in Radioiodine-Refractory Thyroid Cancer. N. Engl. J. Med..

[4] Kiyota N., Schlumberger M., Muro K., Ando Y., Takahashi S., Kawai Y., Wirth L., Robinson B., Sherman S., Suzuki T. (2015). Subgroup Analysis of Japanese Patients in a Phase 3 Study of Lenvatinib in Radioiodine-Refractory Differentiated Thyroid Cancer. Cancer Sci..

[5] Wells S.A., Robinson B.G., Gagel R.F., Dralle H., Fagin J.A., Santoro M., Baudin E., Elisei R., Jarzab B., Vasselli J.R. (2012). Vandetanib in Patients with Locally Advanced or Metastatic Medullary Thyroid Cancer: A Randomized, Double-Blind Phase III Trial. J. Clin. Oncol..

[6] Kreissl M.C., Bastholt L., Elisei R., Haddad R., Hauch O., Jarząb B., Robinson B., Colzani R., Foster M., Weiss R. (2020). Efficacy and Safety of Vandetanib in Progressive and Symptomatic Medullary Thyroid Cancer: Post Hoc Analysis From the ZETA Trial. J. Clin. Oncol..

[7] Elisei R., Schlumberger M.J., Müller S.P., Schöffski P., Brose M.S., Shah M.H., Licitra L., Jarzab B., Medvedev V., Kreissl M.C. (2013). Cabozantinib in Progressive Medullary Thyroid Cancer. J. Clin. Oncol..

[8] Schlumberger M., Elisei R., Müller S., Schöffski P., Brose M., Shah M., Licitra L., Krajewska J., Kreissl M.C., Niederle B. (2017). Overall Survival Analysis of EXAM, a Phase III Trial of Cabozantinib in Patients with Radiographically Progressive Medullary Thyroid Carcinoma. Ann. Oncol..

[9] Cabanillas M.E., Hu M.I., Durand J.-B., Busaidy N.L. (2011). Challenges Associated with Tyrosine Kinase Inhibitor Therapy for Metastatic Thyroid Cancer. J. Thyroid Res..

[10] Paschke L., Lincke T., Mühlberg K.S., Jabs W.J., Lindner T.H., Paschke R. (2018). Anti VEGF-TKI Treatment and New Renal Adverse Events Not Reported in Phase III Trials. Eur. Thyroid J..

[11] Haddad R.I., Schlumberger M., Wirth L.J., Sherman E.J., Shah M.H., Robinson B., Dutcus C.E., Teng A., Gianoukakis A.G., Sherman S.I. (2017). Incidence and Timing of Common Adverse Events in Lenvatinib-Treated Patients from the SELECT Trial and Their Association with Survival Outcomes. Endocrine.

[12] Brose M.S., Worden F.P., Newbold K.L., Guo M., Hurria A. (2017). Effect of Age on the Efficacy and Safety of Lenvatinib in Radioiodine-Refractory Differentiated Thyroid Cancer in the Phase III SELECT Trial. J. Clin. Oncol..

[13] Wirth L.J., Tahara M., Robinson B., Francis S., Brose M.S., Habra M.A., Newbold K., Kiyota N., Dutcus C.E., Mathias E. (2018). Treatment-Emergent Hypertension and Efficacy in the Phase 3 Study of (E7080) Lenvatinib in Differentiated Cancer of the Thyroid (SELECT). Cancer.

[14] Locati L.D., Piovesan A., Durante C., Bregni M., Castagna M.G., Zovato S., Giusti M., Ibrahim T., Puxeddu E., Fedele G. (2019). Real-World Efficacy and Safety of Lenvatinib: Data from a Compassionate Use in the Treatment of Radioactive Iodine-Refractory Differentiated Thyroid Cancer Patients in Italy. Eur. J. Cancer.

[15] Tsang V.H.M. (2019). Management of Treatment-Related Toxicities in Advanced Medullary Thyroid Cancer. Curr. Opin. Oncol..

[16] Lamartina L., Ippolito S., Danis M., Bidault F., Borget I., Berdelou A., Al Ghuzlan A., Hartl D., Blanchard P., Terroir M. (2016). Antiangiogenic Tyrosine Kinase Inhibitors: Occurrence and Risk Factors of Hemoptysis in Refractory Thyroid Cancer. J. Clin. Endocrinol. Metab..

[17] Hui E.P., Ma B.B.Y., King A.D., Mo F., Chan S.L., Kam M.K.M., Loong H.H., Ahuja A.T., Zee B.C.Y., Chan A.T.C. (2011). Hemorrhagic Complications in a Phase II Study of Sunitinib in Patients of Nasopharyngeal Carcinoma Who Has Previously Received High-Dose Radiation. Ann. Oncol..

[18] Onoda N., Tokumoto M., Noda S., Ohira G., Kashiwagi S., Hirakawa K. (2016). A Case of Recurrent Anaplastic Thyroid Cancer Treated by Lenvatinib after Successful Long-Term Multimodal Therapy. Nihon Rinsho Geka Gakkai Zasshi (J. Jpn. Surg. Assoc.).

[19] Haugen B.R., Alexander E.K., Bible K.C., Doherty G.M., Mandel S.J., Nikiforov Y.E., Pacini F., Randolph G.W., Sawka A.M., Schlumberger M. (2016). 2015 American Thyroid Association Management Guidelines for Adult Patients with Thyroid Nodules and Differentiated Thyroid Cancer: The American Thyroid Association Guidelines Task Force on Thyroid Nodules and Differentiated Thyroid Cancer. Thyroid.

[20] Hay I.D., Lee R.A., Davidge-Pitts C., Reading C.C., Charboneau J.W. (2013). Long-Term Outcome of Ultrasound-Guided Percutaneous Ethanol Ablation of Selected “Recurrent” Neck Nodal Metastases in 25 Patients with TNM Stages III or IVA Papillary Thyroid Carcinoma Previously Treated by Surgery and 131I Therapy. Surgery.

[21] Durante C., Haddy N., Baudin E., Leboulleux S., Hartl D., Travagli J.P., Caillou B., Ricard M., Lumbroso J.D., De Vathaire F. (2006). Long-Term Outcome of 444 Patients with Distant Metastases from Papillary and Follicular Thyroid Carcinoma: Benefits and Limits of Radioiodine Therapy. J. Clin. Endocrinol. Metab..

[22] Motzer R.J., Jonasch E., Boyle S., Carlo M.I., Manley B., Agarwal N., Alva A., Beckermann K., Choueiri T.K., Costello B.A. (2021). NCCN Guidelines Insights: Thyroid Carcinoma, Version 1.2021. J. Natl. Compr. Canc. Netw..

[23] Ando Y., Elisei R., Schlumberger M. (2015). Subgroup Analysis according to Differentiated Thyroid Cancer Histology in the Phase 3 (SELECT) Trial of Lenvatinib.

[24] Tahara M., Kiyota N., Hoff A.O., Badiu C., Owonikoko T.K., Dutcus C.E., Suzuki T., Ren M., Wirth L.J. (2021). Impact of Lung Metastases on Overall Survival in the Phase 3 SELECT Study of Lenvatinib in Patients with Radioiodine-Refractory Differentiated Thyroid Cancer. Eur. J. Cancer.

[25] Brose M.S., Smit J., Lin C.-C., Pitoia F., Fellous M., DeSanctis Y., Schlumberger M., Tori M., Sugitani I. (2017). Timing of Multikinase Inhibitor Initiation in Differentiated Thyroid Cancer. Endocr. Relat. Cancer.

[26] Smit J.W.A., Brose M.S., Pitoia F., Lin C.-C., Sugitani I., Alevizaki M., Godbert Y., Aller J., Peeters R.P., Pazaitou-Panayiotou K. (2017). Interim Baseline Characteristics from RIFTOS MKI, a Global Non-Interventional Study Assessing the Use of Multikinase Inhibitors (MKIs) in the Treatment of Patients with Asymptomatic Radioactive Iodine-Refractory Differentiated Thyroid Cancer (RAI-R DTC): A European Subgroup Analysis. Ann. Oncol..

[27] Smit J., Brose M., Lin C.-C., Fellous M., Pitoia F., Sugitani I., Schlumberger M. (2016). Baseline patient characteristics from riftos: A global noninterventional study evaluating the use of multikinase inhibitors for treatment of asymptomatic differentiated thyroid cancer refractory to radioactive iodine (RIFTOS MKI): P3-06-07. Eur. Thyroid J..

[28] Brose M.S., Smit J.W.A., Lin C.-C., Tori M., Bowles D.W., Worden F., Shen D.H.-Y., Huang S.-M., Alevizaki M., Peeters R.P. (2020). 1918P Final Analysis of RIFTOS MKI, a Global, Non-Interventional Study Assessing the Use of Multikinase Inhibitors (MKIs) for the Treatment of Patients with Asymptomatic Radioactive Iodine-Refractory Differentiated Thyroid Cancer (RAI-R DTC). Ann. Oncol..

[29] Sueta D., Suyama K., Sueta A., Tabata N., Yamashita T., Tomiguchi M., Takeshita T., Yamamoto-Ibusuki M., Yamamoto E., Izumiya Y. (2017). Lenvatinib, an Oral Multi-Kinases Inhibitor, -Associated Hypertension: Potential Role of Vascular Endothelial Dysfunction. Atherosclerosis.

[30] Pharmaceutical Interview Forms_Lenvatinib_ver.11. https://image.packageinsert.jp/pdf.php?mode=1&yjcode=4291039M1020.

[31] Yu S.-T., Ge J.-N., Luo J.-Y., Wei Z.-G., Sun B.-H., Lei S.-T. (2019). Treatment-Related Adverse Effects with TKIs in Patients with Advanced or Radioiodine Refractory Differentiated Thyroid Carcinoma: A Systematic Review and Meta-Analysis. Cancer Manag. Res..

[32] Bamias A., Manios E., Karadimou A., Michas F., Lainakis G., Constantinidis C., Deliveliotis C., Zakopoulos N., Dimopoulos M.A. (2011). The Use of 24-H Ambulatory Blood Pressure Monitoring (ABPM) during the First Cycle of Sunitinib Improves the Diagnostic Accuracy and Management of Hypertension in Patients with Advanced Renal Cancer. Eur. J. Cancer.

[33] Maitland M.L., Bakris G.L., Black H.R., Chen H.X., Durand J.-B., Elliott W.J., Ivy S.P., Leier C.V., Lindenfeld J., Liu G. (2010). Initial Assessment, Surveillance, and Management of Blood Pressure in Patients Receiving Vascular Endothelial Growth Factor Signaling Pathway Inhibitors. J. Natl. Cancer Inst..

[34] Ancker O.V., Wehland M., Bauer J., Infanger M., Grimm D. (2017). The Adverse Effect of Hypertension in the Treatment of Thyroid Cancer with Multi-Kinase Inhibitors. Int. J. Mol. Sci..

[35] Zamorano J.L., Lancellotti P., Rodriguez Muñoz D., Aboyans V., Asteggiano R., Galderisi M., Habib G., Lenihan D.J., Lip G.Y.H., Lyon A.R. (2016). 2016 ESC Position Paper on Cancer Treatments and Cardiovascular Toxicity Developed under the Auspices of the ESC Committee for Practice Guidelines: The Task Force for Cancer Treatments and Cardiovascular Toxicity of the European Society of Cardiology (ESC). Eur. Heart J..

[36] James P.A., Oparil S., Carter B.L., Cushman W.C., Dennison-Himmelfarb C., Handler J., Lackland D.T., LeFevre M.L., MacKenzie T.D., Ogedegbe O. (2014). 2014 Evidence-Based Guideline for the Management of High Blood Pressure in Adults. JAMA.

[37] Uy A.L., Simper N.B., Champeaux A.L., Perkins R.M. (2009). Progressive Bevacizumab-Associated Renal Thrombotic Microangiopathy. Clin. Kidney J..

[38] Bollee G., Patey N., Cazajous G., Robert C., Goujon J.-M., Fakhouri F., Bruneval P., Noel L.-H., Knebelmann B. (2008). Thrombotic Microangiopathy Secondary to VEGF Pathway Inhibition by Sunitinib. Nephrol. Dial. Transplant..

[39] Eremina V., Jefferson J.A., Kowalewska J., Hochster H., Haas M., Weisstuch J., Richardson C., Kopp J.B., Kabir M.G., Backx P.H. (2008). VEGF Inhibition and Renal Thrombotic Microangiopathy. N. Engl. J. Med..

[40] Izzedine H., Rixe O., Billemont B., Baumelou A., Deray G. (2007). Angiogenesis Inhibitor Therapies: Focus on Kidney Toxicity and Hypertension. Am. J. Kidney Dis..

[41] Zhang Z.-F., Wang T., Liu L.-H., Guo H.-Q. (2014). Risks of Proteinuria Associated with Vascular Endothelial Growth Factor Receptor Tyrosine Kinase Inhibitors in Cancer Patients: A Systematic Review and Meta-Analysis. PLoS ONE.

[42] Iwasaki H., Yamazaki H., Takasaki H., Suganuma N., Sakai R., Nakayama H., Toda S., Masudo K. (2019). Renal Dysfunction in Patients with Radioactive Iodine-Refractory Thyroid Cancer Treated with Tyrosine Kinase Inhibitors: A Retrospective Study. Medicine.

[43] Takahashi S., Kiyota N., Yamazaki T., Chayahara N. (2016). Phase II Study of Lenvatinib in Patients with Differentiated, Medullary, and Anaplastic Thyroid Cancer: Final Analysis Results. J. Clin. Oncol..

[44] Masaki C., Sugino K., Kobayashi S., Akaishi J., Hames K.Y., Tomoda C., Suzuki A., Matsuzu K., Uruno T., Ohkuwa K. (2020). Urinalysis by Combination of the Dipstick Test and Urine Protein–creatinine Ratio (UPCR) Assessment Can Prevent Unnecessary Lenvatinib Interruption in Patients with Thyroid Cancer. Int. J. Clin. Oncol..

[45] Izzedine H., Massard C., Spano J.P., Goldwasser F., Khayat D., Soria J.C. (2010). VEGF Signalling Inhibition-Induced Proteinuria: Mechanisms, Significance and Management. Eur. J. Cancer.

[46] Takahashi S., Kiyota N., Tahara M. (2017). Optimal Use of Lenvatinib in the Treatment of Advanced Thyroid Cancer. Cancers Head Neck.

[47] Parsa V., Heilbrun L., Smith D., Sethi A., Vaishampayan U. (2009). Safety and Efficacy of Sorafenib Therapy in Patients with Metastatic Kidney Cancer with Impaired Renal Function. Clin. Genitourin. Cancer.

[48] Goto H., Kiyota N., Otsuki N., Imamura Y., Chayahara N., Suto H., Nagatani Y., Toyoda M., Mukohara T., Nibu K.-I. (2018). Successful Treatment Switch from Lenvatinib to Sorafenib in a Patient with Radioactive Iodine-Refractory Differentiated Thyroid Cancer Intolerant to Lenvatinib due to Severe Proteinuria. Auris Nasus Larynx.

[49] Robinson B.G., Paz-Ares L., Krebs A., Vasselli J., Haddad R. (2010). Vandetanib (100 Mg) in Patients with Locally Advanced or Metastatic Hereditary Medullary Thyroid Cancer. J. Clin. Endocrinol. Metab..

[50] Armstrong T.S., Wen P.Y., Gilbert M.R., Schiff D. (2012). Management of Treatment-Associated Toxicites of Anti-Angiogenic Therapy in Patients with Brain Tumors. Neuro-Oncology.

[51] Machiels J.-P.H., Henry S., Zanetta S., Kaminsky M.-C., Michoux N., Rommel D., Schmitz S., Bompas E., Dillies A.-F., Faivre S. (2010). Phase II Study of Sunitinib in Recurrent or Metastatic Squamous Cell Carcinoma of the Head and Neck: GORTEC 2006-01. J. Clin. Oncol..

[52] Eisai (2015). Data on File.

[53] Prescribing Information for LENVIMA (Lenvatinib). http://www.lenvima.com/pdfs/prescribing-information.pdf.

[54] Cabanillas M.E., Takahashi S. (2020). Managing the Adverse Events Associated with Lenvatinib Therapy in Radioiodine-Refractory Differentiated Thyroid Cancer. Head Neck Tumors (HNT).

[55] Spigel D.R., Hainsworth J.D., Yardley D.A., Raefsky E., Patton J., Peacock N., Farley C., Burris H.A., Anthony Greco F. (2010). Tracheoesophageal Fistula Formation in Patients with Lung Cancer Treated with Chemoradiation and Bevacizumab. J. Clin. Oncol..

[56] Blevins D.P., Dadu R., Hu M., Baik C., Balachandran D., Ross W., Gunn B., Cabanillas M.E. (2014). Aerodigestive Fistula Formation as a Rare Side Effect of Antiangiogenic Tyrosine Kinase Inhibitor Therapy for Thyroid Cancer. Thyroid.

[57] Resteghini C., Cavalieri S., Galbiati D., Granata R., Alfieri S., Bergamini C., Bossi P., Licitra L., Locati L.D. (2017). Management of Tyrosine Kinase Inhibitors (TKI) Side Effects in Differentiated and Medullary Thyroid Cancer Patients. Best Pract. Res. Clin. Endocrinol. Metab..

[58] Price D.L., Wong R.J., Randolph G.W. (2008). Invasive Thyroid Cancer: Management of the Trachea and Esophagus. Otolaryngol. Clin. N. Am..

[59] Kaman L. (2011). Management of Esophageal Perforation in Adults. Gastroenterol. Res..

[60] Iwasaki H., Toda S., Murayama D., Kato S., Matsui A. (2021). Relationship between Adverse Events Associated with Lenvatinib Treatment for Thyroid Cancer and Patient Prognosis. Mol. Clin. Oncol..

[61] Verheul H.M.W., Pinedo H.M. (2007). Possible Molecular Mechanisms Involved in the Toxicity of Angiogenesis Inhibition. Nat. Rev. Cancer.

[62] Walraven M., Witteveen P.O., Lolkema M.P.J., van Hillegersberg R., Voest E.E., Verheul H.M.W. (2011). Antiangiogenic Tyrosine Kinase Inhibition Related Gastrointestinal Perforations: A Case Report and Literature Review. Angiogenesis.

[63] Kamba T., McDonald D.M. (2007). Mechanisms of Adverse Effects of Anti-VEGF Therapy for Cancer. Br. J. Cancer.

[64] Date E., Okamoto K., Fumita S., Kaneda H. (2018). Gastrointestinal Perforation Related to Lenvatinib, an Anti-Angiogenic Inhibitor That Targets Multiple Receptor Tyrosine Kinases, in a Patient with Metastatic Thyroid Cancer. Investig. New Drugs.

[65] Stone R.L., Sood A.K., Coleman R.L. (2010). Collateral Damage: Toxic Effects of Targeted Antiangiogenic Therapies in Ovarian Cancer. Lancet Oncol..

[66] Qi W.-X., Sun Y.-J., Tang L.-N., Shen Z., Yao Y. (2014). Risk of Gastrointestinal Perforation in Cancer Patients Treated with Vascular Endothelial Growth Factor Receptor Tyrosine Kinase Inhibitors: A Systematic Review and Meta-Analysis. Crit. Rev. Oncol. /Hematol..

[67] Chen H.X., Cleck J.N. (2009). Adverse Effects of Anticancer Agents That Target the VEGF Pathway. Nat. Rev. Clin. Oncol..

[68] Kitamura M., Hayashi T., Suzuki C., Hirano S., Tateya I., Kishimoto Y., Omori K. (2017). Successful Recovery from a Subclavicular Ulcer Caused by Lenvatinib for Thyroid Cancer: A Case Report. World J. Surg. Oncol..

[69] Harshman L.C., James Yu R., Allen G.I., Srinivas S., Gill H.S., Chung B.I. (2013). Surgical Outcomes and Complications Associated with Presurgical Tyrosine Kinase Inhibition for Advanced Renal Cell Carcinoma (RCC). Urol. Oncol. Semin. Orig. Investig..

[70] Feyerabend S., Schilling D., Wicke C., Stenzl A. (2009). Toxic Dermatolysis, Tissue Necrosis and Impaired Wound Healing due to Sunitinib Treatment Leading to Forefoot Amputation. Urol. Int..

[71] Chapin B.F., Delacroix S.E., Culp S.H., Nogueras Gonzalez G.M., Tannir N.M., Jonasch E., Tamboli P., Wood C.G. (2011). Safety of Presurgical Targeted Therapy in the Setting of Metastatic Renal Cell Carcinoma. Eur. Urol..

[72] Jonasch E., Wood C.G., Matin S.F., Tu S.-M., Pagliaro L.C., Corn P.G., Aparicio A., Tamboli P., Millikan R.E., Wang X. (2009). Phase II Presurgical Feasibility Study of Bevacizumab in Untreated Patients with Metastatic Renal Cell Carcinoma. J. Clin. Oncol..

[73] Powles T., Kayani I., Blank C., Chowdhury S., Horenblas S., Peters J., Shamash J., Sarwar N., Boletti K., Sadev A. (2011). The Safety and Efficacy of Sunitinib before Planned Nephrectomy in Metastatic Clear Cell Renal Cancer. Ann. Oncol..

[74] Silberstein J.L., Millard F., Mehrazin R., Kopp R., Bazzi W., DiBlasio C.J., Patterson A.L., Downs T.M., Yunus F., Kane C.J. (2010). Feasibility and Efficacy of Neoadjuvant Sunitinib before Nephron-Sparing Surgery. BJU Int..

[75] Nunes Q.M., Li Y., Sun C., Kinnunen T.K., Fernig D.G. (2016). Fibroblast Growth Factors as Tissue Repair and Regeneration Therapeutics. PeerJ.

[76] Toda S., Iwasaki H., Murayama D., Nakayama H., Suganuma N., Masudo K. (2021). Invasive Procedures in Patients Undergoing Treatment with Lenvatinib for Thyroid Cancer. Mol. Clin. Oncol..

[77] Yamazaki H., Sugino K., Matsuzu K., Masaki C., Akaishi J., Hames K., Tomoda C., Suzuki A., Uruno T., Ohkuwa K. (2020). Rapid Disease Progression after Discontinuation of Lenvatinib in Thyroid Cancer. Medicine.

[78] Mancuso M.R., Davis R., Norberg S.M., O’Brien S., Sennino B., Nakahara T., Yao V.J., Inai T., Brooks P., Freimark B. (2006). Rapid Vascular Regrowth in Tumors after Reversal of VEGF Inhibition. J. Clin. Investig..

[79] Hasinoff B.B. (2010). The Cardiotoxicity and Myocyte Damage Caused by Small Molecule Anticancer Tyrosine Kinase Inhibitors Is Correlated with Lack of Target Specificity. Toxicol. Appl. Pharmacol..

[80] Shah R. (2016). Cardiovascular Safety of Tyrosine Kinase Inhibitors: Putting Their “QT-Phobia” in Perspective. ADMET DMPK.

[81] Jiang L., Ping L., Yan H., Yang X., He Q., Xu Z., Luo P. (2020). Cardiovascular Toxicity Induced by Anti-VEGF/VEGFR Agents: A Special Focus on Definitions, Diagnoses, Mechanisms and Management. Expert Opin. Drug Metab. Toxicol..

[82] Tocchetti C.G., Gallucci G., Coppola C., Piscopo G., Cipresso C., Maurea C., Giudice A., Iaffaioli R.V., Arra C., Maurea N. (2013). The Emerging Issue of Cardiac Dysfunction Induced by Antineoplastic Angiogenesis Inhibitors. Eur. J. Heart Fail..

[83] Van Marcke C., Ledoux B., Petit B., Seront E. (2015). Rapid and Fatal Acute Heart Failure Induced by Pazopanib. BMJ Case Rep..

[84] Eisai Eisai Announces Publication of Post Hoc Analysis Data of Lenvima® (Lenvatinib) from Phase 3 Select Trial in Certain Patients with Differentiated Thyroid Cancer in the European Journal of Cancer. https://eisai.mediaroom.com/2021-04-29-Eisai-Announces-Publication-of-Post-Hoc-Analysis-Data-of-LENVIMA-R-lenvatinib-from-Phase-3-SELECT-Trial-in-Certain-Patients-with-Differentiated-Thyroid-Cancer-in-the-European-Journal-of-Cancer.

[85] Plana J.C., Galderisi M., Barac A., Ewer M.S., Ky B., Scherrer-Crosbie M., Ganame J., Sebag I.A., Agler D.A., Badano L.P. (2014). Expert Consensus for Multimodality Imaging Evaluation of Adult Patients during and after Cancer Therapy: A Report from the American Society of Echocardiography and the European Association of Cardiovascular Imaging. Eur. Heart J. Cardiovasc. Imaging.

[86] Lenihan D.J., Kowey P.R. (2013). Overview and Management of Cardiac Adverse Events Associated with Tyrosine Kinase Inhibitors. Oncologist.

[87] Mouhayar E., Durand J.-B., Cortes J. (2013). Cardiovascular Toxicity of Tyrosine Kinase Inhibitors. Expert Opin. Drug Saf..

[88] Zarbin M.A. (2018). Anti-VEGF Agents and the Risk of Arteriothrombotic Events. Asia Pac. J. Ophthalmol..

[89] Spivak J.L. (2002). Polycythemia Vera: Myths, Mechanisms, and Management. Blood.

[90] Tam B.Y.Y., Wei K., Rudge J.S., Hoffman J., Holash J., Park S.-K., Yuan J., Hefner C., Chartier C., Lee J.-S. (2006). VEGF Modulates Erythropoiesis through Regulation of Adult Hepatic Erythropoietin Synthesis. Nat. Med..

[91] Bai Y., Li J.-Y., Li J., Zhang B., Liu Y.-H., Zhang B.-Y., Jing J. (2019). Risk of Venous and Arterial Thromboembolic Events Associated with Tyrosine Kinase Inhibitors in Advanced Thyroid Cancer: A Meta-Analysis and Systematic Review. Oncotarget.

[92] Strevel E.L., Ing D.J., Siu L.L. (2007). Molecularly Targeted Oncology Therapeutics and Prolongation of the QT Interval. J. Clin. Oncol..

[93] Lu Z., Wu C.-Y.C., Jiang Y.-P., Ballou L.M., Clausen C., Cohen I.S., Lin R.Z. (2012). Suppression of Phosphoinositide 3-Kinase Signaling and Alteration of Multiple Ion Currents in Drug-Induced Long QT Syndrome. Sci. Transl. Med..

[94] Shumaker R.C., Zhou M., Ren M., Fan J., Martinez G., Aluri J., Darpo B. (2014). Effect of Lenvatinib (E7080) on the QTc Interval: Results from a Thorough QT Study in Healthy Volunteers. Cancer Chemother. Pharmacol..

[95] Ghatalia P., Je Y., Kaymakcalan M.D., Sonpavde G., Choueiri T.K. (2015). QTc Interval Prolongation with Vascular Endothelial Growth Factor Receptor Tyrosine Kinase Inhibitors. Br. J. Cancer.

[96] Grande E., Kreissl M.C., Filetti S., Newbold K., Reinisch W., Robert C., Schlumberger M., Tolstrup L.K., Zamorano J.L., Capdevila J. (2013). Vandetanib in Advanced Medullary Thyroid Cancer: Review of Adverse Event Management Strategies. Adv. Ther..

[97] Abu Rmilah A.A., Lin G., Begna K.H., Friedman P.A., Herrmann J. (2020). Risk of QTc Prolongation among Cancer Patients Treated with Tyrosine Kinase Inhibitors. Int. J. Cancer.

[98] Yang J.-G., Wang L.-L., Ma D.-C. (2018). Effects of Vascular Endothelial Growth Factors and Their Receptors on Megakaryocytes and Platelets and Related Diseases. Br. J. Haematol..

[99] Butt M.I., Bakhsh A.M.K., Nadri Q.J. (2021). Lenvatinib-Induced Multiorgan Adverse Events in Hurthle Cell Thyroid Cancer: A Case Report. World J. Clin. Oncol..

[100] Ye J.Y., Chan G.C.F., Qiao L., Lian Q., Meng F.Y., Luo X.Q., Khachigian L.M., Ma M., Deng R., Chen J.L. (2010). Platelet-Derived Growth Factor Enhances Platelet Recovery in a Murine Model of Radiation-Induced Thrombocytopenia and Reduces Apoptosis in Megakaryocytes via Its Receptors and the PI3-k/Akt Pathway. Haematologica.

[101] Avraham H., Price D.J. (1999). Regulation of Megakaryocytopoiesis and Platelet Production by Tyrosine Kinases and Tyrosine Phosphatases. Methods.

[102] Schutz F.A.B., Je Y., Choueiri T.K. (2011). Hematologic Toxicities in Cancer Patients Treated with the Multi-Tyrosine Kinase Sorafenib: A Meta-Analysis of Clinical Trials. Crit. Rev. Oncol./Hematol..

[103] Zhu C., Ma X., Hu Y., Guo L., Chen B., Shen K., Xiao Y. (2016). Safety and Efficacy Profile of Lenvatinib in Cancer Therapy: A Systematic Review and Meta-Analysis. Oncotarget.

[104] Liu J., Nicum S., Reichardt P., Croitoru K., Illek B., Schmidinger M., Rogers C., Whalen C., Jayson G.C. (2018). Assessment and Management of Diarrhea Following VEGF Receptor TKI Treatment in Patients with Ovarian Cancer. Gynecol. Oncol..

[105] Schmidinger M. (2013). Understanding and Managing Toxicities of Vascular Endothelial Growth Factor (VEGF) Inhibitors. EJC Suppl..

[106] Hescot S., Vignaux O., Goldwasser F. (2013). Pancreatic Atrophy—A New Late Toxic Effect of Sorafenib. N. Eng. J. Med..

[107] Ahmadieh H., Salti I. (2013). Tyrosine Kinase Inhibitors Induced Thyroid Dysfunction: A Review of Its Incidence, Pathophysiology, Clinical Relevance, and Treatment. BioMed Res. Int..

[108] Sanda M., Tamai H., Deguchi H., Mori Y., Moribata K., Shingaki N., Ueda K., Inoue I., Maekita T., Iguchi M. (2011). Acalculous Cholecystitis in a Patient with Hepatocellular Carcinoma on Sorafenib. ISRN Gastroenterol..

[109] Aihara Y., Yoshiji H., Yamazaki M., Ikenaka Y., Noguchi R., Morioka C., Kaji K., Tastumi H., Nakanishi K., Nakamura M. (2012). A Case of Severe Acalculous Cholecystitis Associated with Sorafenib Treatment for Advanced Hepatocellular Carcinoma. World J. Gastrointest. Oncol..

[110] De Lopes G.L., Lima C.M.R. (2007). Emphysematous Cholecystitis in a Patient with Gastrointestinal Stromal Tumor Treated with Sunitinib. Pharmacotherapy.

[111] Gomez-Abuin G., Karam A.A., Mezzadri N.A., Bas C.A. (2009). Acalculous Cholecystitis in a Patient with Metastatic Renal Cell Carcinoma Treated with Sunitinib. Clin. Genitourin. Cancer.

[112] Da Fonseca L.G., Barroso-Sousa R., Sabbaga J., Hoff P.M. (2014). Acute Acalculous Cholecystitis in a Patient with Metastatic Renal Cell Carcinoma Treated with Sunitinib. Clin. Pract..

[113] Nakano K., Suzuki K., Morita T. (2012). Life-Threatening Acute Acalculous Cholecystitis in a Patient with Renal Cell Carcinoma Treated by Sunitinib: A Case Report. J. Med. Case Rep..

[114] Pharmaceuticals and Medical Devices Agency of Japan Summary of Investigation Results—Lenvatinib Mesylate. http://www.pmda.go.jp/files/000222160.pdf.

[115] Nervo A., Ragni A., Gallo M., Ferraris A., Fonio P., Piovesan A., Arvat E. (2020). Symptomatic Biliary Disorders During Lenvatinib Treatment for Thyroid Cancer: An Underestimated Problem. Thyroid.

[116] Tahara M., Brose M.S., Wirth L.J., Suzuki T., Miyagishi H., Fujino K., Dutcus C.E., Gianoukakis A. (2019). Impact of Dose Interruption on the Efficacy of Lenvatinib in a Phase 3 Study in Patients with Radioiodine-Refractory Differentiated Thyroid Cancer. Eur. J. Cancer.

[117] Matsuyama C., Ueda Y., Suzuki S., Fujisawa T., Ito K., Enokida T., Okano S., Tahara M. (2021). Planned Drug Holidays during Treatment with Lenvatinib for Radioiodine-Refractory Differentiated Thyroid Cancer (RR-DTC): A Retrospective Study. Ann. Oncol..

[118] Tahara M., Takami H., Ito Y., Okamoto T., Sugitani I., Sugino K., Takahashi S., Takeyama H., Tsutsui H., Hara H. (2021). Planned Drug Holiday in a Cohort Study Exploring the Effect of Lenvatinib on Differentiated Thyroid Cancer. J. Clin. Oncol..

[119] Shumaker R., Aluri J., Fan J., Martinez G., Pentikis H., Ren M. (2015). Influence of Hepatic Impairment on Lenvatinib Pharmacokinetics Following Single-Dose Oral Administration. J. Clin. Pharmacol..

[120] Brose M.S., Panaseykin Y., Konda B., de la Fouchardiere C., Hughes B.G.M., Gianoukakis A.G., Park Y.J., Romanov I., Krzyzanowska M.K., Binder T. (2020). 426P A Multicenter, Randomized, Double-Blind, Phase II Study of Lenvatinib (LEN) in Patients (pts) with Radioiodine-Refractory Differentiated Thyroid Cancer (RR-DTC) to Evaluate the Safety and Efficacy of a Daily Oral Starting Dose of 18 Mg vs 24 Mg. Ann. Oncol..

[121] Subbiah V., Kreitman R.J., Wainberg Z.A., Cho J.Y., Schellens J.H.M., Soria J.C., Wen P.Y., Zielinski C., Cabanillas M.E., Urbanowitz G. (2018). Dabrafenib and Trametinib Treatment in Patients With Locally Advanced or Metastatic BRAF V600-Mutant Anaplastic Thyroid Cancer. J. Clin. Oncol..

[122] Shah M.H., Wei L., Wirth L.J., Daniels G.A., De Souza J.A., Timmers C.D., Sexton J.L., Beshara M., Nichols D., Snyder N. (2017). Results of Randomized Phase II Trial of Dabrafenib versus Dabrafenib plus Trametinib in BRAF-Mutated Papillary Thyroid Carcinoma. J. Clin. Orthod..

[123] Drilon A., Laetsch T.W., Kummar S., DuBois S.G., Lassen U.N., Demetri G.D., Nathenson M., Doebele R.C., Farago A.F., Pappo A.S. (2018). Efficacy of Larotrectinib in TRK Fusion-Positive Cancers in Adults and Children. N. Engl. J. Med..

[124] Doebele R.C., Drilon A., Paz-Ares L., Siena S., Shaw A.T., Farago A.F., Blakely C.M., Seto T., Cho B.C., Tosi D. (2020). Entrectinib in Patients with Advanced or Metastatic NTRK Fusion-Positive Solid Tumours: Integrated Analysis of Three Phase 1–2 Trials. Lancet Oncol..

[125] Wirth L.J., Sherman E., Robinson B., Solomon B., Kang H., Lorch J., Worden F., Brose M., Patel J., Leboulleux S. (2020). Efficacy of Selpercatinib in RET-Altered Thyroid Cancers. N. Engl. J. Med..

[126] Subbiah V., Hu M.I., Wirth L.J., Schuler M., Mansfield A.S., Curigliano G., Brose M.S., Zhu V.W., Leboulleux S., Bowles D.W. (2021). Pralsetinib for Patients with Advanced or Metastatic RET-Altered Thyroid Cancer (ARROW): A Multi-Cohort, Open-Label, Registrational, Phase 1/2 Study. Lancet Diabetes Endocrinol..

